# Italian onco-haematological patients’ preferences in bad news communication: a preliminary investigation

**DOI:** 10.1186/s12885-021-08181-0

**Published:** 2021-05-17

**Authors:** Ramona Bongelli, Alessia Bertolazzi, Ludovica Piccioni, Roberto Burro

**Affiliations:** 1 Department of Political Science, Communication and International Relations, University of Macerata, Via Don Minzoni 22/A, 62100 Macerata, Italy; 2 Department of Human Sciences, University of Verona, Verona, Italy

**Keywords:** Onco-haematological disease, Truth, Bad news, Patients’ experiences, Patients’ preferences

## Abstract

**Background:**

The manner in which bad news is communicated in oncological contexts can affect patients’ engagement, their coping strategies and therapeutic compliance. Although this topic has been broadly investigated since the nineties, to the best of our knowledge, little has been written about Italian patients’ *experiences* and *preferences* concerning *what* the oncologists should disclose and *how* they should intimate patients about their health conditions in different stages of oncological disease.

**Methods:**

In an attempt to fill this gap, an online self-report questionnaire was administered to a sample of Italian onco-haematological patients. Data were analysed both qualitatively (by a content analysis) and quantitatively (by descriptive analysis and Generalized Linear Mixed Model).

**Results:**

While the majority of patients elected to know the truth during their clinical course, a polarisation between those arguing that the truth be fully disclosed and those claiming that the truth be communicated in a personalised way was observed at the attitude level. Among demographic variables accounted for, *age* seems to most affect patients’ preferences. Indeed, younger Italian patients decidedly reject concealment of the truth, even when justified by the beneficence principle. This result could be a reaction to some protective and paternalistic behaviours, but it could even reflect a relation according to which the more the age increases the more the fear of knowing rises, or an intergenerational change due to different ways of accessing the information. The qualitative analysis of the final open-ended question revealed three main sources of problems in doctor-patient encounters: scarcity of time, absence of empathy and use of not-understandable language that makes it difficult for patients to assume a more active role.

**Conclusions:**

The results of the present study, which represents a preliminary step in the subject investigation, will be deployed for the construction and validation of a more sophisticated questionnaire. Better awareness of the Italian onco-haematological patients’ preferences concerning bad news communication and truth-telling could be useful in adopting more suitable medical practices and improving doctor-patient relationships.

## Background

It is known that truth-telling attitudes and practices on breaking bad news during the doctor-patient encounter have been greatly reformed in the past four decades. These have been shifting from full concealment of bad diagnosis or poor prognosis, especially about life-threatening diseases such as cancer, to acknowledgement of the disclosure of health information as every patient’s right. Primarily in oncological contexts, breaking bad news (cancer diagnosis, negative prognosis, relapse post-surgery or treatment, and so on) can significantly impact the patients’ engagement, coping strategies, and therapeutic compliance. Given the importance of this topic—for its clinical, psychological, and social implications—the disclosure of bad news has been broadly investigated since the nineties with respect to physicians’ and patients’ truth-telling preferences and practices.

Thus, doctor-patient negotiation on delivering bad news has been a relevant object of inquiry, through two main research lines: (a) on the one hand, the studies adopting a “medical-centred perspective”, i.e. those mainly focusing on identification and assessment of clinical protocols for communicating bad news [1–5]; and (b) on the other hand, the studies having a “patient-centred perspective”, i.e. those focusing on the patients’ experiences and preferences regarding “what” to know, i.e. what the oncologists should disclose or cover, and “how” to be informed, i.e. how the oncologists should reveal sensitive information [6–19].

Concerning the first line of investigation, three truth-telling models have been identified: *non-disclosure*, *full disclosure* and *individual disclosure* reflecting different relational, communicational and decision-making styles in the doctor-patient relationship [1]. According to Donovan [1], non-disclosure and full-disclosure models can be framed as a paternalistic relationship between physicians and patients, due to the lack of regard for patients’ preferences concerning the timing and the amount of the bad news to be disclosed. Conversely, the individual disclosure model foresees personalised communication between patients and physicians. It implies that patients differ according to the amount of information and time needed to accept and adjust to bad news. Through a negotiation, patients and physicians should clarify the information the patient prefers to know and the best way to communicate it.

Despite the increasing attention towards these communicative aspects as a result of rising awareness of their importance for clinical outcomes, at the onset of the new millennium, contrasts between the physicians’ general attitude and their routine practice in the clinical setting were noted. By investigating the attitudes of a group of physicians regarding truth-telling to cancer patients, Grassi et al. [20 p. 43] discovered that “while about half the sample indicated that, in principle, patients should always be informed of the diagnosis, only one quarter reported that they always disclosed the diagnosis in practice”.

To simplify the arduous task of revealing unpleasant truths, Baile et al. [3] developed one of the best-known protocols for breaking bad news named SPIKES. SPIKES is the acronym for six strategies which, according to the authors, should prove worthwhile in reducing patients’ distress during oncological encounters and which could be used by doctors: **S**etting up the interview, assessing patients’ **P**erception (i.e. evaluating their ability to understand), obtaining patients’ **I**nvitation (to be informed or not) and waiting for their possible questions, giving **K**nowledge and information by avoiding technical terms and pre-announcing bad news, addressing patients’ **E**motions with Empathic responses, and sharing **S**trategy and Summarising. This protocol, as well as other similar ones (e.g. ABCDE [21], and BREAKS [22]), has enjoyed a consistently positive reputation, and it has been utilised for training medical students in breaking bad news [23–26].

Therefore, where the first line of investigation focuses on how physicians should communicate bad news, the second one pays attention to identifying patients’ preferences. Much research has indeed been carried out with the main purpose of knowing the way in and the extent to which patients prefer to be informed about their cancer, mainly through self-report measures (i.e. questionnaires).^1^ Based on the currently available evidence, it seems fair to suggest that a large majority of patients expect a full disclosure pattern from physicians and pay particular attention to the content of the communication, such as comprehensiveness of the information received or competence of the physicians and, to a lesser extent, to the setting’s aspects, i.e. privacy or time and empathic support [3, 6, 8, 10, 13, 29]. Furthermore, despite the recommendations (see SPIKES), the supportive aspects and physical contact are often evaluated as inappropriate [17]. Similarly, contrary to the expectations, the effects of anticipating bad news (which, at least ideally, should prepare the patient, thus avoiding shocking him/her) do not appear to be significantly associated with a lower degree of patient distress [14].

Although, therefore, much research has been conducted on these topics, most has focused on breaking bad news in general (without explicitly distinguishing between diagnosis and prognosis) and on patient samples mainly from western countries.

On the first aspect, most of these studies deal generically with the communication of bad news, primarily referring to the disclosure of negative diagnoses and disease relapses. Nonetheless, as far as we know, only a few of them explicitly account for the patients’ preferences for communication of undesirable prognosis [14], especially in advanced-stage cancer [30]. This is probably because conveying a bad prognosis is one of the most difficult and complex communicative activities both for patients and for physicians [30–32]. These last, as reported by Rodriguez et al. ([33] p. 219), often have difficulties in discussing prognosis because of “the uncertain progression of disease, varied treatment responses, and differences in how patients want to receive this information”, and because of the onerous task of balancing honesty with hope [34]. Regarding the second point, despite the quantum of research just mentioned, to the best of our knowledge, studies on patients from Southern European countries (and more in general on non-western patients) are, at present, fewer in number^2^; controversial in their results, and, specifically in the Italian context, outdated and mainly focused on patients’ satisfaction [40, 41], demoralisation [42, 43], coping styles [44] etc., i.e. on strictly psychological aspects rather than on communicative preferences.

It is well-known that legal and ethical codes on informed consent and the patient’s right to know have been adopted by many western and eastern countries. Nonetheless, at the practical level, cross-cultural differences seem to resist and influence truth-telling attitudes and acts [3, 45, 46]. This has been observed in the case of Asian countries, such as Japan [47], China [48, 49], Singapore [50, 51]; Middle Eastern countries [52] and Southern European countries, i.e. Greece [53], Spain [54], and Italy [55–57].^3^ These nations appear to be characterised by a protective approach to patients; the acceptance of medical authority regarding the decision to reveal the truth or not; the active role attributed to the patient’s family regarding the choice of fully disclosing the truth; and patients’ preferences for partial disclosure concerning bad news, such as poor prognosis or length of survival. Even though the above-mentioned studies seem to confirm the permanence of stereotyped cross-cultural differences, some others appear controversial in their results [16, 32, 58]. Specifically, in the Italian context, Mauri et al. [9 p. 1529] noted some ambiguity among patients “who mostly want to be informed about diagnosis but are confused about this opportunity: the right to be informed in a timely manner to participate in their care is both desired and avoided; a participatory model of relationship that is less asymmetric is not considered as important”.

Since much uncertainty still exists, specifically, about Italian patients’ preferences, the main objectives of this study are (1) to detect their attitudes to the disclosure of the truth about diagnosis and prognosis, and (2) to consider their implications to enhance the communication of bad news within the patient-physician relationship.

This study, which represents a preliminary step in the subject investigation, could shed some light on a poorly investigated topic, thus improving the knowledge of the Italian onco-haematological patients’ preferences concerning bad news communication and truth-telling. Better awareness of these aspects could be beneficial in adopting more suitable medical practices and improving doctor-patient relationships.

## Method

### Procedures

An exploratory questionnaire made of 20 items has been drafted after five interviews with four physicians (who handle onco-haematological diseases) and two leukaemia patients. The items of the questionnaire (see Table 1 in Section Main  Results) have been chosen to identify some relevant dimensions of doctor-patient interaction and are a starting point for building a subsequent questionnaire.

This first version of the questionnaire was administered online (via LimeSurvey, an open-source survey application [59], which limits any possible risk to patients’ confidentiality) in two different periods to six closed Facebook groups of leukaemia patients, who were numerous enough to be representative. The social media site was chosen to speedily recruit the highest possible number of participants afflicted with onco-haematological diseases and then transition to the construction of a new survey.

In the first section of the present questionnaire some information concerning (a) the subjects eligible to compile the questionnaire, i.e. only the patients; (b) the aims of the research; (c) the contacts of one of the researchers (who could be contacted to receive more detailed information about the study and the questionnaire); (d) how the results will be disseminated (scientific publications, workshops, and conferences); and (e) the references to the European and Italian privacy laws and the protection of personal data have been provided.

Since it was an exploratory questionnaire, no pre-test was performed.^4^

After moderators’ approvals, the questionnaire was launched in the first two Facebook groups:

1. “Lotta alla Leucemia, Linfomi e Mieloma, dona il sangue, regala una speranza”/“Fight against Leukemia, Lymphomas and Myeloma, gives blood, gives hope” (a closed group with 7900 members, who are patients and non-patients), and
2. “Trapiantati di midollo, autotrapianto, leucemie, linfomi e mielomi”/“Transplanted marrow, autograft, leukaemia, lymphoma and myeloma” (a closed group with 3330 members. All of those are patients).

The first administration was closed after 2 weeks. A total of 157 patients agreed to participate and completed the questionnaire. Following a common practice, to have a more representative sample, the second administration was shared with other four groups:

3. “Mieloma multiplo Italia esperienze e news”/“Multiple myeloma, Italy experiences and news” (a closed group with 1300 members),
4. “Linfoma non hodgkin” / “Non-hodgkin’s lymphoma” (a closed group with 3200 members)
5. “Linfoma non hodgkin a grandi cellule B mediastino”/“Non-hodgkin’s lymphoma in large B-cell in the mediastinum” (a closed group with 444 members),
6. “Leucemia mieloide cronica”/“Chronic myeloid leukaemia” (a closed group with 303 members).

The second administration was closed after 2 months. A total of 286 patients agreed to participate and completed the questionnaire. Both the choice to run the survey on the other four Facebook groups and to leave it for a longer time had an impact on sample size by increasing the number of respondents and consequently allowing us to better stratify data.

No reminder during the first and the second administration was needed since every time a patient filled the questionnaire and/or added comments, the link for its compilation reappeared among the news of the Facebook groups. We did not offer any prize as an incentive to compile the survey, since it is not a common practice in Italy and as it can also affect the data, by inducing subjects to give socially desirable answers, thus compromising the data reliability [60]. Furthermore, leukaemia patients also seemed to be highly motivated to participate and share their experiences without any prize.

A total of 443 patients completed the questionnaire during the first and second administration. A total of 60 questionnaires have been rejected for lacking one or more demographic responses (age, gender, and level of education). These variables, used as factors in our analyses, are indeed central; therefore, the inclusion of those questionnaires would not have made sense and, on the contrary, it would have been statistically inappropriate. Thus, the final sample included 383 patients.

Data were analysed both quantitatively (through descriptive statistics and GLMM, Generalized Linear Mixed Models) and qualitatively (through a content analysis).

### Analysis

Descriptive analysis was performed to detect demographic data, patients’ attitudes, and their experiences. Jasp 0.11.10 software was employed.^5^

Qualitative analysis was applied to the final open-ended question of the questionnaire to know patients’ opinions concerning their views on improvement in patient-physician communication. Specifically, a content analysis was performed on the responses, deriving code categories from the text inductively [63–66]. Through an initial scoping review of each answer, a first coder (AB, a health sociologist expert in qualitative research and content analysis) created a codebook, with a description of the code system and examples. The coding categories were identified considering the main critical aspects of the patient-doctor communication which emerged from text data. This pilot coding instrument was discussed by two members of the research team (AB and RB), reviewing and resolving discrepancies. In particular, the coders decided to consider in distinct categories the patients who responded that they had no improvement aspects to report and those who pointed out two or more aspects, not classifiable in the other categories identified. Using the finalised codebook, a second coder (RB, a communication psychologist expert in content analysis) coded 21% of the answers (*n* = 46). Inter-coder reliability was calculated through Cohen’s Kappa, which was on average .83, indicating a high level of reliability.^6^ In order to transform our open-ended responses into nominal-level, we used mlogit.data function of the mlogit package [68] within the R-software (Version 3.6.0, R Core Team, Wien, Austria, 2019).

GLMM (Generalized Linear Mixed Model) was performed, as implemented in the glmer function of the lme4 package [69] within the R-software, to verify if there were significant differences in patients’ responses related to the following demographic variables:

- gender (male, female),
- age (18–30; 31–40; 41–50; 51–60; > 60),
- level of education (junior high school; senior high school; graduate and doctorate).

Every time the variable was dichotomous, therefore, we considered that the statistical distribution was binomial and the link function was “logit”: choice or not choice of a specific response category in interaction with the different levels of the variables under exam. For the multiple comparisons in the post-hoc analysis, we used z-statistic with Bonferroni correction.

The subjects’ identity (ID) has been used since the differences between subjects has been modelled as a random effect. We performed Mixed Model ANOVA Tables via likelihood ratio tests.

The interaction has been limited to two:

- *Gender x Item*: male * level 1 vs. female * level 1; male * level 2 vs. female * level 2 etc.
- *Age x Item*: “18–30” * level 1 vs. “31–40” * level 1; “18–30” * level 1 vs. “41–50” * level 1 etc.
- *Level of education x Item*: junior high school * level 1 vs. secondary school * level 1; junior high school * level 1 vs. degree/graduation and PhD * level 1 etc.

## Main results

In the following sections, the results of our analyses will be present separately.

### Descriptive statistical analysis

Specifically, in this section, we will present some of the main results of the descriptive analysis. A synopsis is present in Table 1.

**Table 1:** Descriptive data

ITEMS	Socio-demographic characteristics	Number of patients and %
**1**	**Gender**	
	Male	104 (27.15%)
	Female	279 (72.85%)
**2**	**Age**	45,32 years (range 18–83 years)
	18–30	74 (19.32%)
	31–40	72 (18.80%)
	41–50	80 (20.89%)
	51–60	106 (27.68%)
	> 60	51 (13.32%)
**3**	**Level of education**	
	Junior high school	73 (19.06%)
	Senior high school	209 (54.57%)
	Graduate / Doctorate	101 (26.37%)
**4**	**Your disease has been diagnosed**	
	more than a year ago	310 (80.94%)
	less than a year ago	32 (8.36%)
	less than six month ago	32 (8.36%)
	missing	9 (2.35%)
**5**	**First diagnosis communicated by**	
	Doctor ward	221 (57.70%)
	Department head	82 (21.41%)
	Family doctor	26 (6.79%)
	Relative	20 (5.22%)
	Another physician	16 (4.18%)
	Doctor undergoing specialized training	15 (3.92%)
	Other healthcare worker	3 (0.78%)
**6**	**The truth about diagnosis must be told**	
	always and in full	192 (50.13%)
	always, but in a personalised way	191 (49.87%)
**7**	**The truth about prognosis must be told**	
	always, but in a personalised way	206 (53.79%)
	always and in full	174 (45.43%)
	never	3 (0.78%)
**8**	**During the first encounter with the doctor you were**	
	accompanied by one or more people	294 (76.76%)
	alone	89 (23.24%)
**9**	**During the subsequent communication encounter with the doctor you were**	
	accompanied by one or more people	288 (75.20%)
	alone	95 (24.80%)
**10**	**Lying to the patient is**	
	not legitimate	321 (83.81%)
	legitimate	62 (16.19%)
**11**	**The doctor who omits a part of the truth to avoid pain to the patient**	
	lies	248 (64.75%)
	does not lie	135 (35.25%)
**12**	**During your illness, you have preferred**	
	to know the truth always	355 (92.69)
	not to know the truth always	28 (7.31%)
**13**	**If you chose “to know always the truth” as the answer to the previous question, indicate the reason**	
	in order to organize my life and that of my relatives	64 (18%)
	because, by character, I am convinced that the truth makes free	28 (8%)
	because knowing the true allow me to better face illness	263 (74%)
**14**	**If you chose “not to know always the truth” as the answer to the previous question, indicate the reason**	
	because, by character, I prefer not to know	2 (6%)
	because during the illness I felt more fragile	9 (31%)
	because not knowing the true allow me to better face illness	17 (63%)
**15**	**How much the way to communicate used by the doctor who diagnosed the disease did affect your way of dealing with your illness?**	
	1 = not at all affected	25 (6.53%)
	2 = slightly affected	19 (4.96%)
	3 = neither affected nor not-affected	66 (17.23%)
	4 = affected	122 (31.85%)
	5 = completely affected	151 (39.43%)
**16**	**During the care pathway as a patient you have been**	
	active and collaborative	299 (78.07%)
	sometimes active and collaborative and sometimes passive and non-collaborative	75 (19.58%)
	passive and non-collaborative	9 (2.35%)
**17**	**For the physicians give bad news is**	
	1 = very difficult	190 (49.61%)
	2 = difficult	88 (22.98%)
	3 = neither difficult nor easy	78 (20.37%)
	4 = easy	14 (3.66%)
	5 = very easy	13 (3.39%)
**18**	**How much are you satisfied with the communication occurred with the doctors during your illness?**	
	1 = not at all satisfied	18 (4.70%
	2 = slightly satisfied	29 (7.57%)
	3 = moderately satisfied	83 (21.67%)
	4 = satisfied	117 (30.55%)
	5 = completely satisfied	136 (35.51%)
**19**	**If you think that something did not function in communicating with your doctor, indicate what:**	
	the use of a too technical and/or too difficult to understand language	79 (20,63%)
	the use of a too simplistic language	22 (5.22%)
	the limited time spent with me during the encounters	124 (32.38%)
	the chosen place for communicating	16 (4.18%)
	the physician’s failure to comprehend the patient’s needs	75 (19.58%)
	the presence of my relatives	19 (4.96%)
	other	48 (12.53%)
**20**	**Do you think that something should be improved in doctor-patient communication?**	open-ended question

As for demographic data, out of 383 patients who fully completed the questionnaire, 279 were women (72.85%) and 104 (27.15%) were men. The mean age was 45.32 years (range 18–83 years). The majority of patients (54.57%) had a secondary school qualification and received the first diagnosis of leukaemia more than a year ago (from when they completed the questionnaire) (80.94%) from the ward physicians (57.70%).

As for patients’ *preferences* concerning the communication of diagnosis and prognosis (items 6–7) and their *attitudes* towards doctors’ omission of truth (items 10–11), their answers are slightly different. The sample was split in half concerning the diagnosis communication (item 6), between those who claimed to prefer a full disclosure (192 = 50.13%) and those who claimed to prefer a personalised one (191 = 49.87%). A somewhat different situation was registered for the communication of prognosis (item 7), which, according to a higher percentage of patients (206 = 53.79%), must always be told but in a personalised way. Regarding doctors’ omission of the truth (item 10), almost all patients (321 = 83.81%) claimed that lying is not legitimate behaviour in physicians. Nonetheless, when the physician tells a lie to avoid the patients’ suffering (item 11), the percentage of those who consider this behaviour unjustifiable, and comparable to telling a lie, fell (248 = 64.75%).

As for the patients’ own *experiences* (items 12–16, 18–20, but also 8–9; see Table 1), the great majority of them (355 = 92.69%) claimed that they always preferred to know the truth (item 12) – because (item 13) it allowed them to better face their illness (263 patients = 74%) – and to have been active and collaborative (299 = 78.07%) during the care pathway (item 16). Nonetheless, most of them also claimed to have been accompanied by one or more people during their first encounter with the doctor (294 = 76.76%), as well as during the subsequent ones (288 = 75.20%) (items 8 and 9, respectively).

Two items (15 and 18; see Table 1) of the questionnaire concerning the patients’ experiences were presented on a Likert scale from 1 to 5 points.

As for item 15, the majority of patients stated that the way used by the physicians to convey information has *affected* (4 point on a Likert scale) or *completely affected* (5 point on a Likert scale) their way of coping with illness (122 patients = 31.85% and 151 patients = 39.43%, respectively), recognising, therefore, a central role to the physicians’ communicative styles in managing oncological encounters and in determining their outcomes.

As for item 18, the majority of patients admitted they were *satisfied* (4 point on the Likert scale = 117 patients = 30.55%) or *completely satisfied* (5 point on the Likert scale = 136 patients =35.51%) with how the physicians communicated with them.

Finally, as for those aspects that have not worked during the communication with the physicians (item 19), patients could select one or more options from a given list. The majority of them indicated: “the limited time spent during the encounters” (124 patients = 32.38%); “the use of a too technical and/or too difficult language” (79 patients = 20.63%); and “the inability of the physician to comprehend the patient’s needs” (75 patients = 19.58%). Few of them also selected: “the use of a too simplistic language” (20 patients = 5.22%); “the presence of relatives” (19 patients = 4.96%); and “the place for communicating” (16 patients = 4.18%). Specifically, for the “presence of relatives”, although this option was selected by a low percentage of patients, those who chose it were mainly the younger ones (9 out of 19 were 18–30 years old).

### Qualitative analysis

The questionnaire concluded with an open-ended question through which the respondents were asked to indicate what could be improved in patient-physician communication. 218 answers were obtained, that is 56,9% of the respondents, among which 162 (74.3%) were females, 56 (25.7%) males, and the median age was 47.12 years.

As can be seen from Tables 2, 12 sub-categories have been detected, which include all the answers of patients. Except for “No suggestion” and “Other”, the remaining 10 sub-categories represent the main aspects that should be improved in doctors’ communication from the interviewees’ viewpoint. These sub-categories were subsequently grouped into three categories: Setting, Empathy and Engagement. The three categories were identified considering the content of the answers and grouping together those sub-categories that, by affinity, were addressed to similar communicative, relational or organisational aspects.

**Table 2:** Results of coded answers to the open-ended question concerning what the patients think it should be improved in doctor-patient communication (item 20)

Categories	Sub-categories	Description	Quotations	n	%	Cohen’s Kappa
Setting	Time	Patient asks for more time during medical consultation	“Time devoted to the patient” (*male, 63 y.o., senior high school*) “Let them have more time for encounters... unfortunately there are many sick people” (*female, 46 y.o., senior high school*)	21	9,6	.726
	Organizationalsetting	Patient complains of lack of personnel and/or referring doctor	“Dealing with a different doctor during each visit does not allow to establish a relationship of knowing and reciprocal trust” (*female, 36 y.o., graduate/doctorate*) “More staff would be needed to give doctors and nurses time to prolong the human relationship with patients. Healthcare workers are often overwhelmed by the amount of work they have to do” (*female, 42 y.o., graduate/doctorate*)	13	6	.367
Empathy	Understanding	Patient would like more understanding	“more understanding of the patient’s reaction” (*female, 58 y.o., senior high school*)“more sympathy” (*female, 44 y.o., senior high school*)	12	5,5	.865
	Humanity	Patient asks for a more sensitive and human relationship with the doctors	“a human relationship with the patient” (*female, 33 y.o., junior high school*) “A little humanity and not being considered as a number” (*female, 31 y.o., graduate/doctorate*)	19	8,7	.891
	Empathy	Patient seeks more empathy from the doctors	“Never, and I mean never, forget to be empathetic, or try to be. If we feel we are understood, we feel more comfortable, more ‘protected’” (*female, 22 y.o., senior high school*) “Empathy. During the oncology treatment I met several doctors, especially in day hospital. Some of them were sensitive, others absolutely not. In an oncology ward where you are in constant contact with death, it is necessary to have more care and attention towards the patient, who is first of all a suffering person” (*female, 33 y.o., graduate/doctorate*)	26	11,9	.889
Engagement	Participation	Patient wants to take an active role in care and she/he requires a personalised approach	“If the patient is prepared and wants to deeply investigate the matter, the doctor must not dismiss him with circumstantial sentences” (*male, 35 y.o., graduate/doctorate*) “That even doctors need to have more faith in our information” (*male, 48 y.o., senior high school*)	18	8,3	.726
	Information	Patient seeks more information and she/he wants to be able to ask questions (i.e. about side effects, disease’s long-term consequences, etc.)	“Give more information about the side effects of the drugs. It is not pleasant to discover them gradually” (*female, 51 y.o., graduate/doctorate*) “Sometimes you ask questions that are obvious to them but for you patient, not having a medical degree, they are not obvious at all, and if they answer you, they do it in a very approximate way not allowing you to understand properly” (*female, 25 y.o., senior high school*)	20	9,2	.877
	Listening	Patient asks attention and to be heard	“Listening, even before the cure” (*female, 56 y.o., graduate/doctorate*) “Yes, listening to the patient and not considering him/her just a case” (*female, 44 y.o., graduate/doctorate*)	10	4,6	1.00
	Language	Patient asks a simple language, free of technical jargon	“Transparency in speaking, even explaining things in more simple terms” (*female, 29 y.o., graduate/doctorate*) “Avoiding too technical language. At the beginning, I struggled to understand what kind of disease I had” (*female, 24 y.o., senior high school*)	14	6,4	1.00
	Openness	Patient wants to know the truth	“Always telling the truth, even if it is hard” (*female, 37 y.o., senior high school*) “Always communicate the truth” (*female, 47 y.o., senior high school*)	7	3,2	1.00
No suggestion	No suggestion	No element is indicated to be improved and/or the doctors’ work is praised	“Nothing. I was assisted in the best way. The faith in God gave me the strength to fight. After 9 years from the transplan I am fine” (*male, 67 y.o., junior high school*) “In my case, I am super satisfied with my doctors, all of them exceptional, special, super skilled and humane people” (*male, 61 y.o., senior high school*)	43	19,7	1.00
Other	Other	Aspects related to two or more dimensions and/or not classifiable in the previous categories	“Purtroppo da migliorare ci sarebbero tante cose...” (*male, 43 y.o., junior high school*) “Unfortunately, it depends on the doctor. I was treated in a children’s hospital when I was 15 and the communication and attention were excellent. Now in adult hospitals I have found countless doctors without any tact or attention to the patient, nor kindness” (*female, 35 y.o., graduate/doctorate*)	15	6,9	.725

The first category Setting (*n* = 34, 15.6%) includes the sub-categories Time (9.6%) and Organisational setting (6%) and refers to features relating to the setting in which the interaction between doctor and patient takes place. Patients highlight problems such as scarcity of time during medical consultation due to the high number of patients or the absence of a referring physician and the difficulties arising from meeting different doctors every time.

The second category Empathy (*n* = 57, 26.1%) regards the empathic relationship between patient and physician, and it comprises the sub-categories Understanding (5.5%), Humanity (8.7%) and Empathy (11.9%). This category focuses on patients’ needs for support in their emotional reactions. In the patients’ view, the communication could be ameliorated if the doctor demonstrated more humanity, sympathy, kindness and helpfulness.

Sub-categories that refer to a more active role of patients have been grouped in a third category (“Engagement”, *n* = 69, 31.7%), which includes the sub-categories Information (9.2%), Engagement (8.3%), Language (6.4%), Listening (4.6%), and Openness (3.2%) and focuses on patients’ involvement in decisions about their condition. These issues directly affect the distribution of knowledge and power within the doctor-patient relationship [70]. Given that technical jargon in a clinical setting can widen the gap in patient-physician understanding and adversely impact patients’ outcomes [71–73], patients interviewed prefer a simpler language. Moreover, patients’ demanding to obtain more information, to be heard, to know the truth, and to receive personalised communication could respond to a need for participation in the decision-making process.

Considering the variables gender and level of education, some differences emerged with respect to the categories identified among those who answered the open-ended question (see Table 3). With regard to gender, chi-squared results indicated that differences between women and men were statistically significant (χ^2^ (4, *N* = 218) = 20.36, *p* < .001). Women reported aspects related to both empathy and patient engagement to a greater extent than men, while men felt that there was nothing to improve in doctors’ communication more than women did. Concerning the level of education (χ^2^ (8, *N* = 218) = 17.83, *p* < .05), those with a higher educational qualification identified patients’ engagement and empathy as the main factors for improving the relationship with doctors. Respondents with a junior high school diploma were more inclined not to indicate any aspect of improvement. Instead, there was no significant association between age and the categories identified (χ^2^ (4, *N* = 218) = 1.21, *p* = .876).

**Table 3:** Respondents to the open-ended question (item 20) by sex, age, and level of education

Categories	n	%	Sex		Age		Level of education		
			Male	Female	≤ 47	≥ 48	Junior high school	Senior high school	Graduate/ Doctorate
Engagement	69	31.7	12 (21.4%)	57 (35.2%)	32 (30,8%)	37 (32.5%)	9 (20.5%)	40 (33.9%)	20 (35.7%)
Empathy	57	26.1	8 (14.3%)	49 (30.2%)	30 (28.8%)	27 (23.7%)	10 (22.7%)	29 (24.6%)	18 (32.1%)
Setting	34	15,6	10 (17.9%)	24 (14.8%)	14 (13.5%)	20 (17.5%)	4 (9.1%)	20 (16.9%)	10 (17.9%)
Other	15	6,9	9 (16.1%)	6 (3.7%)	7 (6.7%)	8 (7%)	4 (9.1%)	7 (5.9%)	4 (7.1%)
No suggestion	43	19,7	17 (30.4%)	26 (16%)	21 (20.2%)	22 (19.3%)	17 (38.6%)	22 (18.6%)	4 (7.1%)
Total	218	100	56 (100%)	162 (100%)	104 (100%)	114 (100%)	44 (100%)	118 (100%)	56 (100%)

### GLMM analysis

The GLMM analysis revealed significant differences concerning patients’ experiences and preferences. Specifically, where *gender* seems to affect mostly patients’ experiences, *age* and *level of education* seem to have a higher influence on patients’ preferences, as shown below in detail.

### Item (5) the first diagnosis was communicated by the department head, ward doctor, family doctor, doctor undergoing specialised training, other physician, other healthcare worker (nurse), or relative

**Fig. 1:**
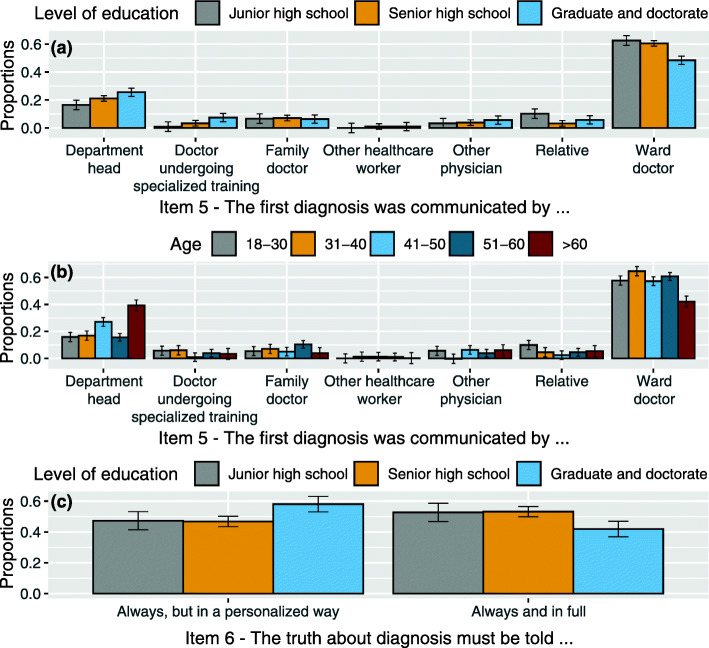
**a** Proportion of responses and 95% confidence intervals related to interaction between item 5 levels (The first diagnosis was communicated by department head, ward doctor, family doctor, doctor undergoing specialised training, other physician, other healthcare worker/nurse, or relative) and levels of education: Junior high school, Senior high school, Graduate and doctorate. **b** Proportion of responses and 95% confidence intervals related to interaction between item 5 levels and age groups: 18–30, 31–40, 41–50, 51–60, > 60. **(c)** Proportion of responses and 95% confidence intervals related to the interaction between item 6 levels (The truth about diagnosis must be told always and in full or always, but in a personalised way, or never, or other) and levels of education: Junior high school, Senior high school, Graduate and doctorate
- interaction between item 5 and *age* (χ^2^(24, *N* = 383) = 57.866, *p* < .001): patients over 60-year-old received more cancer diagnosis from the department head than the other age categories (z = 4.321, *p* < 0.001 if compared with closest 41–50 years old category). Patients who are between 41 and 50 years old received from the department head cancer diagnosis more often than 18–30, 31–40 and 51–60 age categories (z = 3.907, p < 0.001 if compared with closest 31–40 years old category). Patients over 60-year-old less often than the other age categories received the cancer diagnosis from the ward doctor (z = 4.512, p < 0.001 if compared with closest 41–50 years old category). Patients between 18 and 30 years more often received the cancer diagnosis from one of their relatives than the other age categories (z = 2.588, *p* = 0.048 if compared with closest 41–50 years old category). See Fig. 1(b).

As shown in Table 1, frequency analyses of the questionnaires show that the communication of the first cancer diagnosis is generally by the ward doctor (221 = 57.7%) and less frequently by department heads (21.41%), family doctors (6.79%), relatives (5.22%), other physicians (4.18%), doctors undergoing specialised training (3.92%), other healthcare workers (0.78%). Nevertheless, the analysis revealed differences statistically significant related to the following:

- interaction between item 5 levels and *levels of education* (χ^2^(12, *N* = 383) = 21.474, *p* = .043): specifically, graduate patients more often received the first diagnosis from the department head if compared with junior high school patients (z = 2.555, *p* = 0.031) and senior high school patients (z = 2.426, *p* = 0.045); those with low level of education (junior high school certificate) more often received the first diagnosis from one of their own relatives if compared with senior high school certificate (z = 2.499, *p* = 0.037). Patients with junior school certificate and those with senior high school certificate more often received the first diagnosis by the ward doctor if compared with graduate patients (z = 3.111, *p* = 0.006) and senior high school patients (z = 3.020, *p* = 0.007). See Fig. 1(a).

### Item (6) the truth about diagnosis must be told always and in full or always, but in a personalised way, or never, or other

Regarding diagnosis communication, as claimed above (section 3.1), the sample has been split into two parts: on the one hand, those who claimed that the truth must always be told in full (50.1%), and, on the other, those who declared that the truth must always be told, but in a personalised way (49.9%).

The interaction between item 6 and variable *level of education* seems to significantly affect patients’ responses (χ^2^(2, *N* = 383) = 6.715, *p* = .003). Graduate and doctorate patients (more than others) claimed indeed to believe that truth must always be told, but in a personalised way (z = 2.667, *p* = 0.022 if compared with closest junior high school category). See Fig. 1(c).

### Item (7) the truth about prognosis must be told always and in full; always, but in a personalised way; never; other

**Fig. 2:**
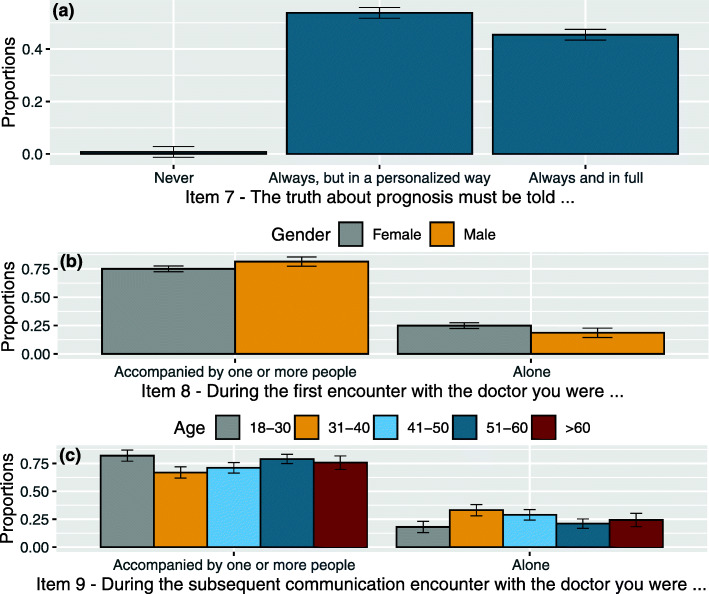
**a** Proportion of responses and 95% confidence intervals related to item 7 levels (The truth about prognosis must be told always and in full; always, but in a personalised way; never; other). **b** Proportion of responses and 95% confidence intervals related to the interaction between item 8 levels (During the first encounter with the doctor, you were alone or accompanied by one or more people) and gender: Female, Male. **c** Proportion of responses and 95% confidence intervals related to the interaction between item 9 levels (During the subsequent communication encounter with the doctor you were alone or accompanied by one or more people) and age groups: 18–30, 31–40, 41–50, 51–60, > 60

Regarding prognosis communication, the analysis did not reveal statistically significant differences related to the interaction between item 7 and *gender*, *age* or *level of education*. Almost all patients claimed to believe that the prognosis must be told (χ^2^(2, *N* = 383) = 366.145, *p* < .001), and the difference with those who claimed that it must be communicated always and in full and those who claimed that it must be communicated in personalised way is not statistically significant. See Fig. 2(a).

### Item (8) during the first encounter with the doctor, you were alone or accompanied by one or more people

A significant number of patients stated they had gone to the first visit not alone, but accompanied by one or more people. The analysis revealed statistically significant differences related exclusively to the interaction between item 8 and *gender* (χ^2^(1, N = 383) = 3.933, *p* = .047). A significantly higher number of male patients claimed to have gone accompanied by one or more relatives or friends to the first medical encounter if compared with female patients (z = 2.292, *p* = 0.043). On the contrary, more female than male patients claimed they went alone to the first medical encounter (z = 2.377, *p* = 0.034). See Fig. 2(b).

### Item (9) during the subsequent communication encounter with the doctor you were alone or accompanied by one or more people

**Fig. 3:**
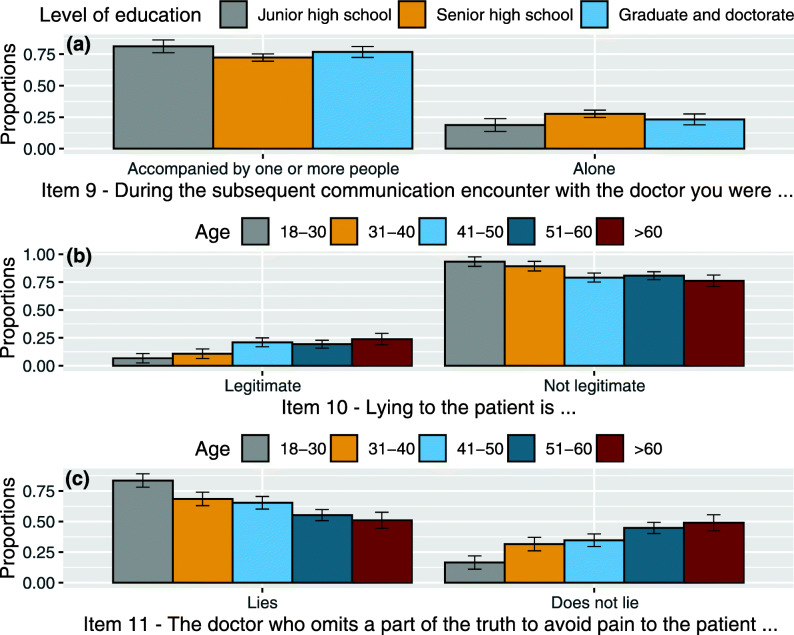
**a** Proportion of responses and 95% confidence intervals related to the interaction between item 9 levels (During the subsequent communication encounter with the doctor you were alone or accompanied by one or more people) and levels of education: Junior high school, Senior high school, Graduate and doctorate. **b** Proportion of responses and 95% confidence intervals related to the interaction between item 10 levels (Lying to the patient is legitimate or not legitimate) and age groups: 18–30, 31–40, 41–50, 51–60, > 60. **c** Proportion of responses and 95% confidence intervals related to the interaction between item 11 levels (The doctor who omits a part of the truth to avoid pain to the patient lies or does not lie) and age groups: 18–30, 31–40, 41–50, 51–60, > 60

Similar to what happened for the first encounter, for the subsequent ones, the majority of patients stated they went with one or more people. Nonetheless, the analysis revealed statistically significant differences related to the following:

- interaction between item 9 levels and *age* (χ^2^(4, *N* = 383) = 11.810, *p* = .019): younger patients (18–30) more often claimed to have been accompanied by one or more people during medical visits if compared with patients between 31 and 40 and 41–50 years (z = 2.780, *p* = 0.027 taking into consideration the closest 41–50 years category). Patients between 51 and 60 stated having been more often accompanied if compared with 31–40 years category (z = 2.678, *p* = 0.037). See Fig. 2(c).
- interaction between item 9 levels and *level of education* (χ^2^(2, *N* = 383) = 5.925, *p* = .050): patients with a low level of education (junior high school certificate) claimed to have been accompanied to medical encounters after the first one more than those with senior high school levels of education (z = 2.481, *p* = 0.039). See Fig. 3(a).

### Item (10) lying to the patient is legitimate or not legitimate

In a statistically significant way, the majority of patients claimed that lying to the patient is not legitimate. Specifically, the interaction between item 10 and variable *age* significantly affects such a response (χ^2^(4, N = 383) = 20.068, *p* < .001). While the younger patients (18–31) claimed that telling a lie to the patients is not a legitimate behaviour, with increasing age, the percentage of those who believe that lying is legitimate also increases. In terms of statistical significance, on the one hand, there are the younger 18–30 and 31–40 years categories, and, on the other hand, there are the other older years categories (z = 2.788, *p* = 0.026 taking into account the two closest categories, for each of the two age groups, 31–40 and 51–60). See Figu. 3(b).

### Item (11) the doctor who omits a part of the truth to avoid pain to the patient lies or does not lie

Similar to our observation for the previous item, also in this case, the majority of patients claimed that the doctor who omits a part of the truth to avoid causing suffering to the patient is telling a lie.

**Fig. 4:**
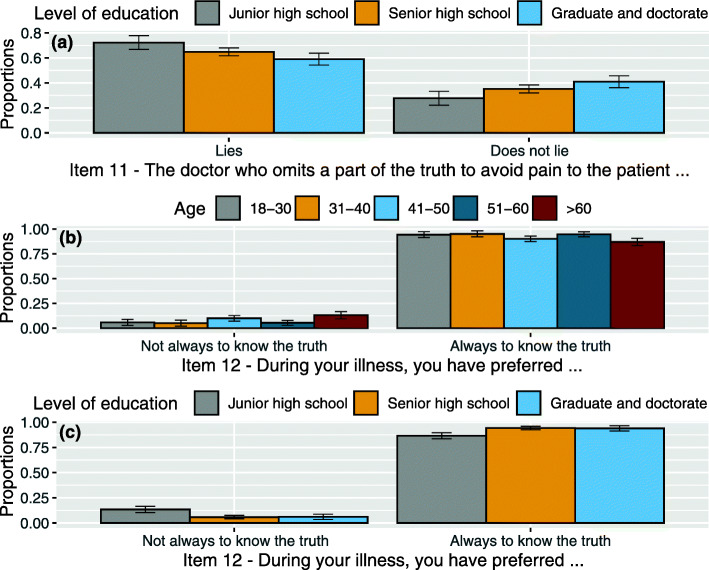
**a** Proportion of responses and 95% confidence intervals related to the interaction between item 11 levels (The doctor who omits a part of the truth to avoid pain to the patient lies or does not lie) and levels of education: Junior high school, Senior high school, Graduate and doctorate. **b** Proportion of responses and 95% confidence intervals related to the interaction between item 12 levels (During your illness, you have preferred always to know the truth or not always to know the truth) and age groups: 18–30, 31–40, 41–50, 51–60, > 60. **c** Proportion of responses and 95% confidence intervals related to interaction between item 12 levels and levels of education: Junior high school, Senior high school, Graduate and doctorate

The difference between those patients who claimed that omitting is the same as lying and those who claimed that omitting is different from lying is statically significant and related to the following:

- interaction between item 11 and *age* (χ^2^(4, *N* = 383) = 37.636, *p* < .001): for the younger patients (more than the older ones) omitting is the same as lying. The younger patients, more than the older ones, consider omitting part of the truth to be the same as lying. The more the age increases, the more the number of those who consider omitting and lying as the same decreases. In terms of statistical significance, there are two differences, the first between 18 and 30 and all other categories (z = 3.621, *p* = 0.001 calculated between 18 and 30 and the closest 31–40) and the second between 31 and 40/41–50 and 51–60/> 60 years categories (z = 2.602, *p* = 0.046 calculated between 41 and 50 and the closest 51–60). See Fig. 3(c).
- interaction between item 11 and *level of education* (χ^2^(2, *N* = 383) = 5.936, *p* = 0.05): patients with junior high school certificate considered omitting part of the truth to be the same as lying if compared with graduate and doctorate category (z = 2.542, *p* = 0.033). See Fig. 4(a).

### Item (12) during your illness, you have preferred always to know the truth or not always to know the truth

In a statistically significant way, the majority of patients claimed that during their illness they have always preferred to know the truth. The observed differences are related to the following:

- interaction between item 12 and *age* (χ^2^(4, *N* = 383) = 9.353, p = 0.05): the older patients (> 60) claimed they not always preferred to know the truth if compared with younger categories (z = 2.644, *p* = 0.041 between > 60 and the closest 18–30 years category). See Fig. 4(b).
- interaction between item 12 and *level of education* (χ^2^(2, *N* = 383) = 9.381, *p* = 0.01): patients with lower levels of education (junior high school certificate) have more often claimed that they have not always preferred to know the truth (z = 2.448, *p* = 0.043 if compared with the closest graduated and doctorate category). See Fig. 4(c).

### Item (16) during the care pathway as a patient you have been active and collaborative or passive and non-collaborative or sometimes active and collaborative and sometimes passive and non-collaborative

**Fig. 5:**
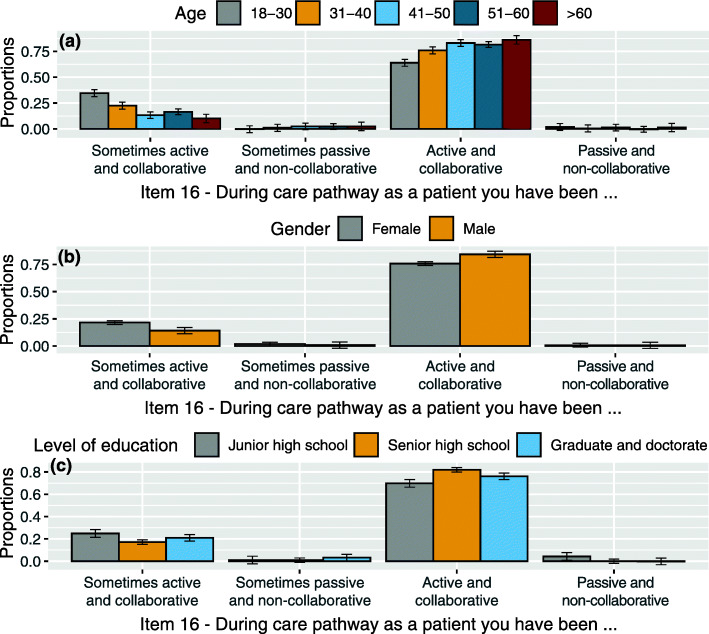
**a** Proportion of responses and 95% confidence intervals related to the interaction between item 16 levels (During care pathway as a patient you have been active and collaborative or passive and non-collaborative or sometimes active and collaborative and sometimes passive and non-collaborative) and age groups: 18–30, 31–40, 41–50, 51–60, > 60. **b** Proportion of responses and 95% confidence intervals related to the interaction between item 16 levels and gender: Female, Male. **c** Proportion of responses and 95% confidence intervals related to the interaction between item 16 levels and levels of education: Junior high school, Senior high school, Graduate and doctorate
- interaction between item 16 and *gender* (χ^2^(3, *N* = 383) = 10.765, *p* = 0.013): more male patients claimed to have been active and collaborative compared to female patients (z = 2.399, *p* = 0.033). Female patients more often claimed that they were active and collaborative sometimes only (z = 2.309, *p* = 0.042). See Fig. 5(b).
- interaction between item 16 and *level of education* (χ^2^(6, N = 383) = 15.436, *p* = 0.017): a higher number of patients with higher level of education claimed to have been active and collaborative when compared to junior high school level (z = 2.434, *p* = 0.045 between junior high school and the closest graduate/doctorate level of education). See Fig. 5(c).

The majority of patients claimed to have been active and collaborative for the main part of the care pathway. The analysis revealed differences statistically significant related to:

- interaction between item 16 and *age* (χ^2^(12, *N* = 383) = 53.072, *p* < 0.001): the more age increases, the more the patients claimed to have been active and collaborative (z = 3.332, *p* = 0.004 between 18 and 30 and the closest 31–40 years category). Younger patients (18–31) in statistically significant way claimed to have been active and collaborative sometimes only (z = 3.299, *p* = 0.005 if compared with the closest 31–40 years category). Independent of age, the patients claiming to have been passive and non-collaborative for most of the care pathway are very few in number. See Fig. 5(a).

## Discussion

The present study is a contribution to the ongoing discussions about patients’ preferences for knowing the truth about their health conditions. Particularly, the objective of the paper was to identify onco-haematological Italian patients’ preferences on breaking bad news, regarding the communication of both the diagnosis and the prognosis.

The results of this study revealed an *ambiguous stance* of Italian patients.

At the practical level, concerning their personal experience, a large majority of the sample chose to know the truth during their clinical course. Despite this, at the attitude level, regarding the way diagnosis and prognosis should be communicated, a polarisation was noted between those who argue for full disclosure and those who prefer to know the truth in a personalised way. As for diagnosis, the individual model is preferred by patients with a higher level of education than those with less education. Concerning the disclosure of prognosis, instead, the desire for an individualised communication increases within the sample, without any significant difference for demographic factors.

Moreover, the legitimisation of concealing the truth presents a dichotomous choice among Italian patients. On the one hand, the majority of the respondents indicate that physicians are not entitled to lie to patients, on the other hand, if the concealment of the truth can avoid causing suffering to patients, the attitudes of the respondents towards the legitimisation of a non-disclosure behaviour increase, especially in older patients. Indeed, Italian younger patients appear to be more inclined to claim a full disclosure style from medical staff, by decidedly rejecting the concealment of the truth, justified by beneficence.

The findings concerning the factor “age” are consistent with previous studies showing that younger patients would seek more engagement in the decision-making process [53, 56, 74–77]. According to Fujimori and Uchitomi [8], seven of the eight studies examined through a systematic literature review found that younger patients prefer receiving as much information as possible. In general, an explanation of the truth attitudes of younger patients could be related to a stance of sureness, courage and ruthlessness which is believed to exist in young people [53]. Meanwhile, Italian younger patients appear to be subjected to protective attitudes and behaviours from relatives and physicians. For example, this study found that, more often than older patients, younger patients have been accompanied by one or more relatives during medical encounters, and they received the communication of diagnosis more frequently from a relative rather than a physician.

Likewise, our data suggest that older people prefer an individual communication style to a full disclosure model, particularly as far as the communication of prognosis or the omission of information to protect the patient are concerned. There are some likely explanations for the differences between young and older patients. A possible reason for these attitudes may be the growing level of fear and apprehension with increasing age. Meanwhile, the young patients’ clear request for truth could be a reaction to some protective and paternalistic behaviours. This hypothesis seems to be consistent with our data according to which the younger patients, more than the older ones, consider the presence of relatives during medical encounters as a possible communicative barrier. Another explanation for these findings might be related to an intergenerational change taking place between old and young patients. The latter would prefer a full disclosure, by supporting the idea that gathering as much information as possible is a requirement for their own self-determination and autonomy. The older patients’ request for personalised communication may be explained as a demand for greater centrality of the patient as well. Nevertheless, a different motivation could be detected in the desire of older patients not to know all the truth. In other terms, the individual communication model would give to the physician a discretion regarding how much truth to disclose to patients. Although Donovan [1] assumes that the individual model is the best approach to truth-telling practice, it seems possible that in certain cultural contexts such a pattern could reproduce a paternalistic relationship within the doctor-patient encounter.

Actually, many studies demonstrated that Italian physicians and patients have been steeped in an enduring tradition of paternalism for a long time. This paternalistic approach, marked by physicians’ control, family-centred interaction and patients’ disposition to accept the medical control, was reflected in many truth-telling attitudes and practices. Regarding traditional practices of concealment of the truth by physicians, a qualitative research on Italian medical students about the contents conveyed by the hidden curriculum has highlighted that students were exposed to a culture of emotional detachment and partial unveiling of the information to patients, in order to protect them from the harm of knowing too much [78]. Besides, prior studies have noted that Italian physicians believed that not all the truth should be disclosed to the patient and that the latter would not want to know the truth; consequently, a section of the physicians did not break bad news in practice [20, 79, 80]. Indeed, a retrospective survey on caregivers of Italian patients who died of cancer has shown that a small portion of patients (37%) had received information regarding diagnosis and only 13% of people were informed about negative prognosis [56].

Regarding the role of the relatives, further studies have observed that a protective approach of the family is a factor that could explain different attitudes and behaviours towards truth-telling, as is the case in nations traditionally centred on family and community values, such as Eastern [16, 81, 82] and South European countries [83, 84], among which stands Italy [41].

Concerning patients’ disposition to accept medical control, some researches have revealed that individuals living in Southern European Catholic countries show less willingness to be informed on poor prognosis and end-of-life decision making [57, 85]. Comparing these results to the findings of the current study, it can be seen that Italian patients surveyed indicated the rise of a changed attitude towards the patient-physician relationship. The qualitative analysis showed that Italian patients would like to know the whole truth, to be heard, to obtain more information, and to take part in the decision-making process. In contrast to researches that have demonstrated that patients affected by life-threatening diseases prefer to entrust control of the encounter to the physician [86, 87], this study reveals that oncological patients, especially the youngest and highly educated, demand an active role and symmetrical communication.

In this investigation, there are several limitations. The study relies on a convenience sample, which does not allow these results to be generalised. Moreover, the survey is focused on a single type of cancer, and the stage of the disease is not known. The informants are members of patients’ Facebook groups; hence, they could be more active and engaged patients than others. Therefore, the relations identified could vary in significance over different patient populations. Further data collected by a probabilistic sample would be required to determine more accurately how age, educational level or different properties could affect patients’ preferences about the delivery of the truth.

Despite the severe limitations of convenience samples, these findings can enhance the understanding of Italian patients’ preferences.

The results of the present study and specifically those coming from the analysis of the final open-ended item of the questionnaire (see Section 3.2) will be used for the construction and validation of a more sophisticated questionnaire. The new version of the questionnaire will include questions that concern: cancer type, the stage of cancer when diagnosed, the specialize doctor charged with communicating diagnosis and prognosis, etc. (lacking from the present version). It could be administered in presence not only to a sample of Italian onco-haematological patients but also to patients suffering from other types of cancer in order to test if there were significant dissimilarities related to solid or non-solid-type tumours. Furthermore, it could be interesting to adopt a cross-cultural perspective to assess if the above-mentioned contextual differences endure over time or if, on the contrary, as Mauri et al. [9 p. 1527] argued some years ago that something is changing also in “those countries with a well-known attitude of non-disclosure, such as Italy”.

Given the importance of the topic for the patients’ wellbeing, future studies could also focus on pursuing not only descriptive results but also practical changes. In order to attain such a goal, it could be interesting to analyse surveys results and present them to a group of patients and health practitioners and ask them their experiences and recommendations on improving doctor-patient communication around giving the diagnosis and discussing prognosis.

## Conclusion

The main aims of our paper were to investigate attitudes of Italian onco-haematological patients to the disclosure of the truth of diagnosis and prognosis, and to take into consideration their implications to enhance the communication of bad news within the patient-physician relationship.

As for patients’ preferences, the descriptive analysis showed some differences concerning the communication of diagnosis and prognosis. Indeed, although almost all of the patients agreed on the need to be informed, i.e. to know the truth of diagnosis and prognosis, the percentage of those who argued that the physicians have to disclose the truth in a personalised way, and not in full, increased in the case of prognosis (suggesting some difficulties in managing possible time-left information, consistent with the results of Harding et al. [57]).

On its part, the GLMM analysis revealed significant differences mainly related to the variables *age* and *level of education.* While the younger patients rejected the concealment of the truth, even when it is justified by the beneficence principle, the older ones and those with higher levels of education argued to consider doctors’ omissions—with the non-maleficence principle as a reason—as a behaviour different from lying. Furthermore, patients with higher levels of education agreed in believing that physicians have to communicate also the truth about diagnosis in a personalised way.

The qualitative analysis of the last item of the questionnaire gave us some suggestions concerning the aspects the patients consider important to improve the communication of bad news. These aspects can be reduced to three main dimensions on which doctors should pay more attention: time and setting management, care of the empathic aspects of the relationship, and greater availability towards patients’ requests to be informed, heard, etc. since that can enable them to play a more active role in the decision-making process concerning their health. Therefore, if, on the one hand, the Italian patients seem to have reached a new awareness about the importance of being informed (compared to the past and to the results, for example, of Mauri et al. [9]), on the other, they also believe that the empathic aspects are equally important (consistent with the suggestions of SPIKES and similar protocols).

The results of this preliminary study allowed us to know both the preferences of a sample of Italian onco-haematological patients concerning bad news communication and truth-telling and their opinions (based on their own experience) about how medical practice should change in order to improve doctor-patient relationships. From a pragmatic perspective, this study represents the first step in developing a more suitable tool for knowing Italian oncological patients’ experiences and preferences when they face bad news.

## Data Availability

The datasets analysed during the current study are available from the corresponding author on reasonable request.

## Abbreviation

**SPIKES**: Stand for the six strategies which, according to Baile et al. (2000), should be useful in reducing patients’ distress during oncological encounters. S (setting), P(patient’s perception), I (patient’s invitation), K (giving knowledge to the patient), E (emphatic responses), S (strategies and summary).
**GLMM**: Stands for Generalized Linear Mixed Model
**ANOVA**: Stands for Analysis Of Variance

## Footnotes

1 Many of these questionnaires try to determine the impact of socio-demographical (age, sex, level of education, and countries of origin), and psychological variables (e.g. levels of depression or attachment styles [27, 28]) on these preferences.

2 Actually, more recently, a lot of research has been conducted in Eastern countries and Asian countries (see for example [8, 24, 35–39]).

3 “Three countries with the lowest percentage of respondents who would wish to know about time left were Italy, Spain and Portugal. It has been suggested that in traditional Mediterranean Catholic countries, patients are more likely to reject autonomy and that the medical establishment may have a greater paternalism” ([57] p. 2303).

4 Actually, the two patients, who were interviewed, viewed and completed the questionnaire, giving us their favorable opinion regarding clarity and comprehensibility of the items.

5 Jasp is a free multi-platform open-source statistics package ([61, 62]).

6 According to Landis and Koch [67], Kappa statistics < 0.00 indicate a poor strength of agreement; 0.00–0.20 slight agreement; 0.21–0.40 fair; 0.41–0.60 moderate; 0.61–0.80 substantial; 0.81–1.00 almost perfect.
